# Occupational Stressors and Safety Behaviour among Oil and Gas Workers in Kuwait: The Mediating Role of Mental Health and Fatigue

**DOI:** 10.3390/ijerph182111700

**Published:** 2021-11-07

**Authors:** Anwar S. Alroomi, Sherif Mohamed

**Affiliations:** School of Engineering and Built Environment, Griffith University, Brisbane 4222, Australia; s.mohamed@griffith.edu.au

**Keywords:** safety behaviour, mental health, fatigue, responsibilities towards family, work–family interface/conflict, living environment

## Abstract

This paper provides an examination of direct and mediated relationships among occupational stressors (responsibilities towards family and living environment), mental health (anxiety and depression), fatigue (physical and mental fatigue), and safety behaviour (safety compliance and safety participation). In this cross-sectional study, data were collected by means of a questionnaire among oil and gas workers (foreign employees working at a remote oil and gas field site located in Kuwait), during a two-month period (November–December 2018). Regression analyses (bivariate and hierarchical), carried out on 387 responses, were employed to test the links between occupational stressors, mental health, fatigue, and safety behaviour in the hypothesised model. The results provide support for the direct relationship in the model, in that both responsibilities towards family and living environment predicted safety behaviour participation. Further, the results provide partial support for the mediated relationships in the model, as mental health and fatigue were found to mediate the relationship of responsibilities towards family and living environment with safety participation behaviour. It is concluded that occupational stressors have a negative effect on safety behaviour, while mental health and fatigue can operate as risk factors. Given this, it is recommended that organisations need to enhance remote oil and gas workers’ safety behaviour by encouraging them to effectively balance their stress, mental health, and level of fatigue. This can be achieved by actions such as promoting spirituality, boosting workers’ resilience, providing recreational facilities and encouraging communications.

## 1. Introduction

In the oil and gas industry, safety is a highly important issue and a procedural problem. It is considered a high-risk industry because of the nature of the work [1]. Workplace accidents are important to investigate because of their profound effect on human life and efforts to reduce them are urgently required [2]. Therefore, in some regions, the industry is acknowledged for its high levels of accidents and injuries, especially those related to psychological injuries [3]. Psychological injury is a broad term that refers to any form of mental ill-health caused by work stress.

According to research [4] stressors are a major component of stress. In the work context, occupational stress is defined as a negative emotional experience generated from demanding work conditions [5]. Stressors can be organisational, individual or environmental [6]. Workers in oil fields are frequently exposed to stressful conditions or permanent physical pressure. Additionally, long-term employment in an isolated location contributes to occupational stress levels [7]. It is worth noting that occupational stress is a feature of offshore life that originates from common sources but also includes the interface between job and family, helicopter travel and the offshore living environment [8].

This study builds on the concept of remoteness. It involves and explores the framework developed by Alroomi and Mohamed [9], where the context was oil and gas workers in Kuwait, the bulk of whom come from diverse Asian countries. These workers are typically relocated to a remote production site in a region foreign to them. They work in such remote sites for an extended period that varies from weeks to months, occasionally lasting a year or longer. Due to the long isolation period, both onshore and offshore subgroups were assumed to face similar hardship and were considered to be working remotely if their work status fits this study’s concept of remoteness.

Bjerkan [10] compared the differences in work-related variables among Norwegian workers onshore and offshore. In an analysis of offshore workers’ perceptions of external influences on work performance, the separation from family and friends was revealed as a significant effect. In addition, being away from home for extended periods can cause relationship stresses. Therefore, these stressors were worthy of investigation in this research to identify their effects on mental health, fatigue, and safety.

Cooper and Sutherland [11] investigated the occupational stressors in the oil and gas industry by focusing on those who worked 14 days offshore followed by a 14-day onshore break. Seven sources of occupational stress that affected North Sea offshore oil workers were identified: (1) relationships at work and at home, (2) site management problems, (3) factors intrinsic to the job, (4) the uncertainty element of the work environment, (5) living in the environment, (6) safety and (7) the interface between job and family. Similarly, Chen, Wong [12] studied 561 Chinese offshore oil workers who worked four weeks offshore followed by a four-week rest onshore. Nine factors of occupational stress were identified: (1) the interface between job and family, (2) career and achievement, (3) safety, (4) management problems and employee relations (incorporated under one label), (5) the physical environment of workplace, (6) the living environment, (7) managerial role, (8) ergonomics and (9) organisational structure.

When analysing the stressors from the two abovementioned studies, only the ‘living environment’, the ‘interface between job and family’ and ‘relationships at work and home’ were found to be related to this study’s concept of remoteness and are the stressors of interest in this study.

### 1.1. Selected Occupational Stressors

The first occupational stressor is responsibilities towards family, a variable (stress factor construct) adopted from the ‘interface between job and family’ factor identified in other research [12]. Responsibilities towards family is a combination of ‘relationships at work and home’ and the ‘interface between job and family’—two factors identified by [11]. This is also integral to research into the work–family interface or conflict in different contexts, in which workers were exposed to high-stress environments [13]. Responsibilities towards family affect a worker’s personal relationships with their family. In addition, responsibilities towards family concern the interference of the family with work or vice versa. A study conducted on university professors discovered that work–family conflict positively correlates with job distress and turnover intentions [14].

The living environment is the second occupational stressor in this study. Similarly to the first stressor, this variable was adopted from both [11,12] and is related to the camp in which workers in this study lived remotely. Many variables are relevant to such shared living which workers might consider a source of stress: privacy, noise, air circulation and camp facilities. Further, the living environment in remote occupations affects work performance. For instance, working and living in a poor environment (i.e., with uncomfortable temperature, lighting, noise, staff density and a minimal degree of privacy) can adversely affect a construction project manager’s work performance [15].

### 1.2. Study Aim, Research Gap and Conceptual Model

**Figure 1:**
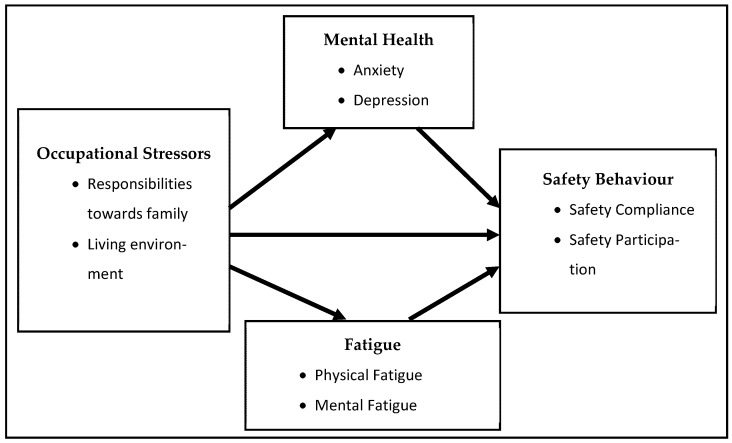
Research conceptual model.

This study aims to investigate the direct relationship between workplace occupational stressors in remote areas and the safety behaviour of workers, and the indirect relationship as mediated by their mental health and fatigue levels. Figure 1 presents the conceptual model of the study.

To the authors’ best knowledge, no prior study has investigated this combination of variables in the oil and gas industry. Offshore work can simply mean that workers are being isolated from family; however, the present study addresses a unique research gap. It focuses on foreign workers having a much longer period of isolation (one year, sometimes extended to two) that includes individuals’ separation from their family, friends, and country. This study’s unique contribution arises from both the conceptual model and the study context. It is important to raise such concerns in a work context with multiple occupational stressors and extended isolation.

### 1.3. Occupational Stressors—Safety Behaviour Relationship

A highly stressful work environment was found to negatively affect human safety [16,17]. The occupational stressors led to lower levels of safety compliance and safety participation and increased the frequency of injuries and near misses [18]. In the oil and gas industry, stress at work can reduce workers’ safety and increase the likelihood of occupational injury [19]. A cross-sectional study in a chemical processing plant, concluded that the perception of role overload (an indication of perceived job stress) was associated with an increased propensity to work unsafely [20]. A study demonstrated that the work–family conflict and interface directly affected predicting safety compliance and safety participation to a significant extent [21]. Similarly, studies [22,23] documented that family interference is negatively linked to safety behaviour. In addition, living conditions are a primary factor responsible for reducing accidents and incidents that require ongoing monitoring and improvements [24].

On the basis of the above discussion, this paper hypothesises that occupational stressors predict safety behaviour, as follows:

**Hypothesis** **1** **(H1).** *Responsibilities towards family predicts both types of safety behaviour (compliance and participation);*

**Hypothesis** **2** **(H2).** *Living environment predicts both types of safety behaviour (compliance and participation).*

### 1.4. Mental Health as a Mediating Variable

#### 1.4.1. Occupational Stressors—Mental Health Relationship

Numerous offshore studies have indicated that occupational stress could predict mental health issues [12,25,26,27]. Cooper and Sutherland [11] discovered that stresses in relationships at work and home are a risk factor for decreased overall wellbeing, free-floating anxiety, depression, and somatic anxiety. Conversely, the stress interface between job and family was positively associated with poor mental health but no such significant association was found with the living environment [28]. However, the living environment was a risk factor for decreased overall wellbeing, free-floating anxiety and phobic anxiety because the requirements of shared living—sleeping quarters, lack of privacy and disturbance by others—each exacerbate the offshore living environment [11].

Moreover, many research studies have documented the effect of work and family interference on psychological health and distress [29,30,31,32]. Work–family conflict positively correlated with depression [33].

#### 1.4.2. Mental Health—Safety Behaviour Relationship

Mental health (i.e., anxiety and depression) induced an increase in accidents or near-miss accidents among professional truck drivers [34]. Depression predicted safety perceptions among UK hospital nurses [35]. A study in the oil and gas industry, determined that psychological distress was negatively associated with employees’ safety behaviour (compliance and participation) [36]. In the construction industry, the use of a non-resident workforce is a common practice; anxiety and depression predicted accident rates and was linked to greater risk of injuries [37,38].

Due to the above reasons, we hypothesise that if a relationship between occupational stressors and safety behaviour exists, it is mediated by mental health, as follows:

**Hypothesis** **3** **(H3).** *Responsibilities towards family predicts safety behaviour via mental health;*

**Hypothesis** **4** **(H4).** *Living environment predicts safety behaviour via mental health.*

### 1.5. Fatigue as a Mediating Variable

#### 1.5.1. Occupational Stressors—Fatigue Relationship

Fatigue highly correlates with poor sleep quality and is characterised by cognitive decline and impairment; therefore, individuals suffering from long-term stress are more likely to report sleeping difficulties [39]. Work–family conflict is related to a higher level of fatigue [40] and lower sleep quality [41]. Similarly, work–family conflict affects sleep quality [42], increases sleep disturbance [43] and is associated with sleep deficiencies [44]. Further, a higher work–family interface was associated with greater workplace cognitive failure and difficulties [45]. In addition, a study among offshore oil and gas workers claimed that both offshore and onshore workers experience sleep-associated social dysfunction [19]. A later study discovered offshore workers reporting higher levels of anxiety, sleep-related problems and workloads [46].

#### 1.5.2. Fatigue—Safety Behaviour Relationship

Fatigue (physical fatigue and mental fatigue) is a major concern to nurses’ work and can threaten their safe practice [47]. In the oil and gas industry, fatigue is a risk factor that affect safety and is frequently linked to accidents [8,48]. Mental fatigue could predict safety compliance behaviour outcomes of oil production workers [49]. Among construction workers, fatigue is positively associated with rates of error [50], and negatively associated with workers’ sense of feeling safe at work [51].

On the basis of the reasons described above, we hypothesise that if a relationship between occupational stressors and safety behaviour exists, it is mediated by fatigue levels, as follows:

**Hypothesis** **5** **(H5).** *Responsibilities towards family predicts safety behaviour via fatigue levels;*

**Hypothesis** **6** **(H6).** *Living environment predicts safety behaviour via fatigue levels.*

## 2. Materials and Methods

### 2.1. Study Setting and Participants

Data for this article comes from a cross-sectional study investigating workplace occupational stressors, mental health, fatigue and safety behaviour among individuals who were working in Kuwaiti oil and gas projects and residing on camp next to their workplace. The projects are located away from major cities, with a low level of available services and infrastructure. Workers work either on oilfields or offshore platforms an extended period that varies from weeks to months, occasionally lasting a year or longer. Due to the long isolation periods, both onshore and offshore subgroups were assumed to face similar hardship and were considered to be working remotely as their work status fitted this study’s concept of remoteness. Workers were welcomed to participate only if they were a non-Kuwaiti (foreign) worker and working at a remote oil and gas field. Participation was voluntary and informed consent was obtained, both of which were managed through direct contact with the oil and gas companies operating in Kuwait and their project’s main contractor. Company representatives assisted the researcher in distributing the survey to the workers. The respondents were asked to return their manually completed survey to the same member of staff that gave it to them. The completed surveys were collated and sorted into envelopes respective of each department. Next, responses were entered in an electronic database and rechecked for accuracy. The researcher only received fully completed surveys to combat the possibility of any missing data. A total of 387 participants completed the survey during a 2-month period (November–December 2018).

### 2.2. Questionnaire Design and Development

The questionnaire developed consists of four main sections (occupational stressors, mental health, fatigue, and safety behaviour). In this study, responsibilities towards family and living environment are the independent variables, mental health and fatigue are the mediators and safety behaviour is the dependent variable. The questionnaire was modified based on the results of a pilot study, where a number of items were deleted. Scales are presented in detail in Table 1.

The first section measures occupational stressors, which was represented by two main variables responsibilities towards family (RTF) and living environment (LE). The scale for both of these variables comprised eight items. All items were scored on a 5-point scale (1 = no stress to 5 = high stress; i.e., the higher the score, the greater the stress). An example is: “Due to work and living away from your home country, please indicate to what degree the following statements currently represents a source of stress for you: Leaving my wife/partner to cope with difficulties or making decisions alone. (1 = Definitely is not a source, 2 = Maybe is not a source, 3 = Neutral, 4 = Maybe is a source, 5 = Definitely is a source).”

The second section measures mental health. A total of 17 items were used to measure the level of anxiety (ANX) and depression (DPR). All items were scored on a 5-point scale (1 = never to 5 = always; i.e., the higher the score, the higher the level of anxiety and depression, the poorer mental health). An example is: “During work or after work hours, please indicate how often you feel this statement applies to you in the last two weeks including today: I find it difficult to have the initiative to do things. (1 = Never, 2 = Rarely, 3 = Sometimes, 4 = Often times, 5 = Always).”

The third section measures the fatigue level. A total of 15 items were used to measure workers physical fatigue (PFT) and mental fatigue (MFT). All items were scored on a 5-point scale (1 = not at all to 5 = very high; i.e., the higher the score, the higher the level of fatigue). An example is: “During work or after work hours, please indicate how often you feel this statement applies to you in the last two weeks including today: Having difficulty concentrating. (1 = not at all to 5 = very high).”

The fourth and final section measures safety behaviour. A total of 15 items were used to measure compliance safety behaviour (SFC) and participation safety behaviour (SFP). All items were scored on a 5-point scale (1 = strongly disagree to 5 = strongly agree; i.e., the higher the score, the higher the level of safety behaviour, compliance and participation). An example is: “In the context of work, please indicate your level of agreement for each statement below: I do not neglect safety, even when in a rush. (1 = Strongly Disagree, 2 = Disagree, 3 = Neutral, 4 = Agree, 5 = Strongly Agree).”

All of the scales for the mental health, fatigue and safety behaviour sections were valid and widely used scales. The pilot study was used to validate the content and reliability of the instruments as the items were slightly modified to suit the oil and gas safety context (see Table 1).

### 2.3. Data Analysis

In the first stage, the dataset was screened and demonstrated an acceptable level of normality without outliers. A limit of 2.58 (0.01 significance level) was used for the skewness and kurtosis. For outliers, a cut-off (z-value = 4.00) was used along with comparing the mean values with 5% trimmed, where the difference between them is not greater than 0.20. The results of SD and SE of the mean indicated that the mean values could be used as representative of the population. A one-way analysis of variance (ANOVA) test was performed on the dataset to determine whether the difference in the perceptions of these respondent groups was statistically significant. The results indicated that the dataset could be treated as a single sample without removing any variables and thus it was considered suitable for further analysis. Consequently, all variables were retained for subsequent analyses.

The second stage starts with examining the scale reliability for the developed scales by employing Cronbach’s alpha. The acceptance level of Cronbach’s alpha for all model constructs indicated that the model constructs accurately and consistently captured their relevant meaning. The pilot study was also used to validate the content and reliability of the instrument. Next, the exploratory factor analysis (EFA) was employed to assess the validity of each measurement scale, and this revealed the number of factors that belonged to each construct. Harman’s single-factor test was conducted to examine the common method variance (CMV). The results suggested that the CMV was not a concern in this study. A confirmatory factor analysis (CFA) was used to confirm the validity of factors derived from the EFA analysis. The CFA results confirmed the EFA findings, with a few amendments, including deleting variables and omitting one factor from the responsibilities towards family construct.

In this research, the model proposes that a set of independent variables influences the dependent variable through other variables (called mediators). According to [68], a mediator effect exists in a particular model if four mediation conditions are met. These conditions are conducted by performing a series of regression analyses, as follows:

1. The independent variable (IV) predicts the mediator variable (M) (Path A);
2. The IV predicts the dependent variable (DV) (Path C);
3. The M predicts the DV (Path B);
4. The indirect effect of the IV on the DV through the M is significant (Path A × B).

To execute the regression analysis for the first three mediation conditions, two regression analyses—bivariate and hierarchical—were applied using the SPSS Version 25. These analyses were conducted to determine the effect sizes, 𝑅^2^ values, the coefficients (b value and beta weight) and significance tests. In addition, the results of the hierarchical regression were explored to assess whether the mediators were either partially or fully mediating the relationships between the factors. Based on [69], full mediation indicates that the independent variable no longer predicts the DV when the mediator is introduced. Partial mediation occurs when the mediator is introduced but the relationship between the IV and DV retains significance; however, it reduced in absolute size and still did not equal 0.

The fourth condition was explored using the PROCESS approach, which is an extension modelling tool that can be added to the SPSS. PROCESS was developed by Preacher and Hayes [70] and provided a bootstrapped Sobel Test of the indirect effect (Path A × B) as a primary advantage.

## 3. Results

### 3.1. Study Setting and Participants

The 387 participants were made up of 295 of Indian origin, with the reminder having various Asian and African origins. A total of 43% of participants were between 30 and 39 years old. Most reported that they were either married or living with a partner (80.4%). A considerable number of workers had returned from their home country just 1 month earlier (39%), while more than 1/4 had not been back to their home country for more than a year. A large proportion of the respondents appeared to spend their days off in camp (77.8%). Demographic characteristics of the sample are shown in Table 2.

### 3.2. Mediation Role of Anxiety

#### 3.2.1. Anxiety Mediates the Relationship between Responsibilities towards Family and Safety Compliance

**Figure 2:**
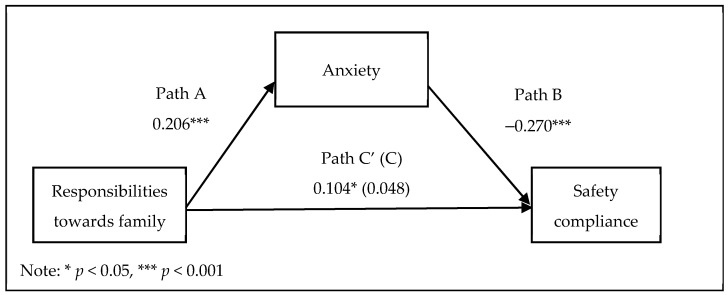
Mediation of the relationship between responsibilities towards family and safety compliance by anxiety.

As Figure 2 illustrates (represented by the standardised regression coefficients), regression analyses were performed to evaluate each component of the hypothesised mediation model. It was found that responsibilities towards family significantly predicted anxiety (Path A), b = 0.211, BCa 95% CI [0.110, 0.311], *p* < 0.001. In addition, the direct effect of responsibilities towards family on safety compliance was not significant (Path C), b = 0.039, BCa 95% CI [−0.042, 0.119], *p* = 0.345, which indicated that the second mediation condition was not met. Specifically, responsibilities towards family accounted for 0.2% of the variance in safety compliance.

When anxiety was added to the regression analysis, responsibilities towards family and anxiety collectively accounted for 7.2% in safety compliance overall, F(2, 384) = 14.88, *p* < 0.001. The proposed mediator—anxiety—significantly predicted safety compliance (Path B), b = −0.212, BCa 95% CI [−0.289, −0.134], *p* < 0.001, and uniquely accounted for 7% of the variance in safety compliance. In addition, the effect of responsibilities towards family on safety compliance became significant after anxiety was added to the regression analysis (Path C’), b = 0.083, BCa 95% CI [0.004, 0.163], *p* < 0.05.

A PROCESS analysis was conducted and revealed that responsibilities towards family significantly predicted safety compliance through its relationship with anxiety (Path A × B), b = −0.0446, BCa 95% CI [−0.077, −0.020]. Further, there was −0.8% of overall change (𝑅^2^) in safety compliance resulting from the relationship between responsibilities towards family and anxiety. However, the second mediation condition was not met, and thus, anxiety failed to be a mediator in the relationship between family responsibilities and safety compliance.

#### 3.2.2. Anxiety Mediates the Relationship between Responsibilities towards Family and Safety Participation

**Figure 3:**
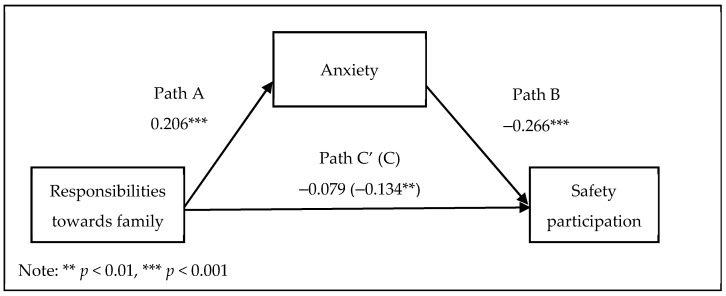
Mediation of the relationship between responsibilities towards family and safety participation by anxiety.

As Figure 3 illustrates and Table 3 enumerates, the relationship between responsibilities towards family and safety participation was mediated by anxiety. The proposed mediation model fully mediated the relationship between responsibilities towards family and safety participation as the direct effect of responsibilities towards family on safety participation was not significant.

#### 3.2.3. Anxiety Mediates the Relationship between Living Environment and Safety Behaviour

As shown in Table 4, the total effect of the living environment on safety compliance was not significant. Thus, the relationship between the living environment and safety compliance was not mediated by anxiety. On the other hand, anxiety mediated the relationship between the living environment and safety participation. In addition, in the proposed mediation model, the relationship between the living environment and safety participation was fully mediated by anxiety as the direct effect of the living environment on safety participation was not significant.

### 3.3. Mediation Role of Depression

#### 3.3.1. Depression Mediates the Relationship between Responsibilities towards Family and Safety Behaviour

As shown in Table 5, the total effect of responsibilities towards family on safety compliance was not significant. Thus, the relationship between responsibilities towards family and safety compliance was not mediated by depression. Contrarily, depression mediated the relationship between responsibilities towards family and safety participation. Results confirmed that in the proposed mediation model, depression fully mediated the relationship between responsibilities towards family and safety participation as the direct effect of responsibilities towards family on safety participation was not significant.

#### 3.3.2. Depression Mediates the Relationship between Living Environment and Safety Behaviour

As shown in Table 6, the total effect of the living environment on safety compliance was not significant. Thus, the relationship between the living environment and safety compliance was not mediated by depression. On the other hand, depression mediated the relationship between the living environment and safety participation. In the proposed mediation model, depression fully mediated the relationship between the living environment and safety participation as the direct effect of the living environment on safety participation was not significant.

### 3.4. Mediation Role of Physical Fatigue

#### 3.4.1. Physical Fatigue Mediates the Relationship between Responsibilities towards Family and Safety Behaviour

As shown in Table 7, the total effect of responsibilities towards family on safety compliance was not significant. Thus, the relationship between responsibilities towards family and safety compliance was not mediated by physical fatigue. Diversely, physical fatigue mediated the relationship between responsibilities towards family and safety participation. The proposed mediation model fully mediated the relationship between responsibilities towards family and safety participation as the direct effect of responsibilities towards family on safety participation was not significant.

#### 3.4.2. Physical Fatigue Mediates the Relationship between Living Environment and Safety Behaviour

As shown in Table 8, the total effect of the living environment on safety compliance was not significant. Thus, the relationship between the living environment and safety compliance was not mediated by physical fatigue. On the other side, physical fatigue mediated the relationship between the living environment and safety participation. The proposed mediation model fully mediated the relationship between the living environment and safety participation as the direct effect of the living environment on safety participation was not significant.

### 3.5. Mediation Role of Mental Fatigue

#### 3.5.1. Mental Fatigue Mediates the Relationship between Responsibilities towards Family and Safety Behaviour

As shown in Table 9, the total effect of responsibilities towards family on safety compliance was not significant. Thus, the relationship between responsibilities towards family and safety compliance was not mediated by mental fatigue. In contrast, the relationship between responsibilities towards family and safety participation was mediated by physical fatigue. The proposed mediation model fully mediated the relationship between responsibilities towards family and safety participation as the direct effect of responsibilities towards family on safety participation was not significant.

#### 3.5.2. Mental Fatigue Mediates the Relationship between Living Environment and Safety Behaviour

As shown in Table 10, the total effect of the living environment on safety compliance was not significant. Thus, the relationship between the living environment and safety compliance was not mediated by mental fatigue. However, the relationship between the living environment and safety participation was mediated by mental fatigue. The proposed mediation model fully mediated the relationship between the living environment and safety participation as the direct effect of the living environment on safety participation was not significant.

## 4. Discussion

This cross-sectional study identified the direct and mediated relationships among occupational stressors (responsibilities towards family and living environment), mental health (anxiety and depression), fatigue (physical fatigue and mental fatigue), and safety behaviour (safety compliance and safety participation). The results suggest that both occupational stressors (responsibilities towards family and living environment) only have a direct and negative influence on safety participation behaviour; thus, H1 and H2 are partially supported.

Regarding the mediating role of mental health, the analysis demonstrated that mental health mediates the relationship between occupational stressors and safety participation. However, it did not mediate the relationship between occupational stressors and safety compliance due to both responsibilities towards family and the living environment not correlating with safety compliance; thus, H3 and H4 are partially supported.

Similarly to mental health, fatigue only mediates the relationship between occupational stressors and safety participation. Based on this finding, H5 and H6 are partially supported.

### 4.1. Direct Relationship between Occupational Stressors and Safety Behaviour

As predicted, the occupational stressors researched in this study (i.e., responsibilities towards family and the living environment) influenced safety behaviour. The results indicated that workers who considered these occupational stressors as potential sources of stress confirmed that they could negatively and significantly affect their safety behaviour and even threaten their lives. It is widely recognised that the majority of accidents are caused by a failure to perform safety behaviour [71,72,73,74]. Further, the negative effect of stress on safety behaviour has been documented in many studies; for example, a recent study claimed that workplace stress causes fatigue among workers, which negatively affects their safety behaviour [75]. Stress significantly influences human behaviours [76] and is highly related to workplace injury incidents [77,78].

Several studies on stress and workplace safety highlight the importance of considering specific work-related stressors, rather than overall stress, when examining workplace safety [79,80]. The effect of work–family conflict (in this study, encapsulated by the value of, responsibilities towards family) has a stronger influence on safety behaviour than other stressors [81]. This may be because the workers are apart from their families as a consequence of working remotely. In this study, responsibilities towards family had a direct and negative influence on safety participation behaviour—a result that was supported by [82], who similarly reported a negative relationship between work–family conflict and safety participation. Likewise, in other recent studies, researchers discussed that work–family conflict had a significant direct effect on predicting safety compliance and safety participation [21] and was negatively linked to employee safety behaviour [22]. Further, work–family conflict can increase the risk of occupational injury [83]. Similarly, the living environment had a direct and negative influence on safety participation behaviour. This finding was congruent with [24], who claimed that living conditions were a significant factor in reducing accidents and required ongoing monitoring and improvements, and [15], who stated that working and living in a poor environment can negatively affect work performance.

Both responsibilities towards family and the living environment were associated with safety participation, which was supported by [84], who claimed that sources of job stress (i.e., occupational stressors) could cause work overload and a lack of participation. However, the results did not indicate an association between these two stressors and safety compliance—a result that contradicted [85], who claimed that stress reduces compliance with safety regulations and causes workers to undertake unsafe practices. An explanation for this finding might be that some workers could deny stress or confirm safe behaviour in questionnaires and thus underreport occupational stressors. Moreover, safety compliance reflected the core procedures that workers must follow to maintain workplace safety, whereas safety participation referred to workers’ participating in activities to improve their safety behaviour. Therefore, workers would likely need to be externally motivated to participate or engage in this additional task.

Workers in a remote or highly stressful work environment tend to lose their motivation because of the influence of occupational stressors; that is, stressed workers are not willing to engage or participate in improving their safety because the stress has induced a loss of motivation. Similarly, a leading safety behaviour study demonstrated that safety participation had a significant positive relationship with motivation, which had further indicated an increase in safety participation but not in safety compliance [65]. Moreover, it is logical that workers in a stressful working environment would be more likely to ignore safety participation because it includes voluntary behaviours than safety compliance, which describes mandatory behaviours. This could explain why the study stressors only correlated with participation and not compliance.

### 4.2. Mediating Role of Mental Health on the Relationship between Occupational Stressors and Safety Behaviour

As predicted, mental health mediated the relationship between occupational stressors and safety behaviour. Specifically, anxiety and depression fully mediated the associations of (responsibilities towards family and the living environment) with (safety participation behaviour). This indicates that mental health could be a negative resource to lower the engagement level of workers in safety participation and increase the adverse effect of occupational stressors on safety behaviour. Similarly, a study on fly-in-fly-out (FIFO) workers showed that reactions to stressors led to anxiety and fatigue, which in turn reduced workers’ performance capacities, including reaction times and judgement, and increased the probability of errors [86]. Further, the results did not indicate any mediation role for mental health factors in the relationship between occupational stressors and safety compliance because the relationship was not statistically significant (as explained previously in Section 4.1).

According to these findings, workers who considered responsibilities towards family as a source of stress reported a higher level of anxiety and depression and were less engaged in safety participation. This finding is congruent with the results of [23], that psychological distress (i.e., anxiety and depression) mediated the relationship between work–family conflict and injuries. Work–family conflict is a potential source of stress that can detrimentally affect wellbeing and behaviour [87]. In addition, work–family conflict was positively linked to depression [33], greater psychological distress [31] and job distress and turnover intentions [14]. The relationship between work–family conflict and psychological health could be explained by the underlying stress caused by extended exposure [88]. In the oil and gas industry, the findings completely supported the literature. The stress interface between job and family pressures is positively associated with poor mental health [28]. Further, it is a risk factor for decreased overall wellbeing, free-floating anxiety, depression and somatic anxiety [11].

Similarly to responsibilities towards family, workers who identified the living environment as a source of stress or experienced problems adapting to the camp reported higher levels of anxiety and depression and were less engaged in safety participation. In previous studies, the living environment was found to decrease overall wellbeing, heighten free-floating anxiety and phobic anxiety [11] and increase psychological distress [89]. Workers claimed these symptoms were predominantly a consequence of the lack of privacy, crowding and disturbance by others.

A study among Chinese offshore workers documented that the overall stress of the living environment was associated with adverse health outcomes as a consequence of the lack of privacy and disturbances from shared living [12]. However, these results contradict others [28], where it was argued that no such significant association was found between the living environment and poor mental health. One explanation for this difference in findings is that the latter study [28] included a four-week isolated period, whereas in the present study a much longer isolation period was documented. Since the workers were expatriate, the separation period varied from months to years and included separation from the workers’ home country. This separation can either restrict the workers’ contracts or influence their decision to save money for the future [90].

Overall, the full mediation role of anxiety and depression as found in this study indicated that responsibilities towards family and the living environment are no longer predictors of safety participation when either anxiety or depression are introduced to the model.

### 4.3. Mediating Role of Fatigue on the Relationship between Occupational Stressors and Safety Behaviour

In this study, fatigue was considered a critical mediator in explaining the influence of occupational stressors on workers’ safety behaviour. As predicted, fatigue levels mediated the relationship between occupational stressors and safety behaviour. Specifically, physical and mental fatigue fully mediated the relationship of (responsibilities towards family and the living environment) with (safety participation). This result indicates that fatigue could be a negative resource to lower workers’ level of engagement in safety participation and increase the adverse effect of occupational stressors on safety behaviour. This result aligns with earlier findings from the UK’s Royal Navy, which demonstrated that cognitive failure mediated the link between stress and accidents [91]. Moreover, it was proven that workplace stress causes fatigue among workers, which negatively affects their safety behaviour [75].

Further, this result was consistent with research [86] regarding the construction industry, which claimed that reactions to stressors led to anxiety and fatigue, which in turn reduced performance capacities, including reaction times and judgement, and increased the probability of errors. Similarly, work pressures led workers to focus their attention on completing their work and increased workers’ tendency to engage in unsafe acts [20]. Further, the results did not indicate any mediation role for fatigue factors in the relationship between occupational stressors and safety compliance because the relationship was not statistically significant (as explained and addressed previously in Section 4.1).

According to these findings, workers who claimed responsibilities towards family as a source of stress reported higher levels of physical and mental fatigue and were less engaged in safety participation. This is supported by [81] who claimed that work and family conflict generated stress that prevented workers from achieving optimal concentration, focus and effort for their work, which reduced their work engagement. Therefore, the significant association between work–family conflict and safety outcomes was a result of workplace cognitive failure. According to another study [85], emotional stress decreases construction workers’ attention and causes them to disregard safety behaviours. In addition, a strong correlation was found between work–family conflict and daily fatigue [92,93].

Similarly to responsibilities towards family, workers who considered the living environment a source of stress or experienced difficulties in adapting to the camp reported a higher level of physical and mental fatigue and were less engaged in safety participation. This is congruent with [94] who also researched the oil and gas industry and claimed that living in shared accommodation camps or crowded dormitories with no privacy or personal space for a long duration caused mental health issues and fatigue. According to researchers of Norwegian offshore workers, half of the sample reported difficulties in sharing cabins and were troubled by snoring, which caused sleeping problems and led to fatigue [95]. Sleep deprivation is a common challenge expressed by workers in shared living quarters [96].

Occupational stress and the safety climate predict fatigue-related behaviour [97]. In addition, occupational fatigue is related to accidents and injuries [98,99]. Nonetheless, in a study by [100] regarding the construction industry, it was claimed that sleep problems were associated with fatigue and both were linked to workplace injuries and accidents. Further, cognitive failure negatively affected safety compliance and safety participation at the bivariate level [22].

Overall, the full mediation role of physical fatigue and mental fatigue indicate that responsibilities towards family and the living environment are no longer predictors of safety participation when either physical fatigue or mental fatigue are present.

### 4.4. Theoretical Implications

The present study fills an important void and contributes to theories in many ways, especially in the occupational safety literature, occupational health psychology and research on complex industries. In complex industries such as the oil and gas sector, where working conditions are stressful, organisations may need to focus on the stress arising from workers’ responsibilities towards their family and the living environment to improve workers’ mental health, fatigue levels and safety behaviour, in particular, safety participation. Previously, researchers who examined the relationship among these variables paid less attention to the importance of mental health and fatigue levels in describing intermediating processes in the link. Accordingly, by investigating the intermediating role of mental health and fatigue levels, we can provide an elaborate explanation on how being or feeling stressed affects safety participation at work beyond basic safety compliance. Thus, examining the role of mental health and fatigue levels in describing the association is essential.

### 4.5. Practical Implications

This paper may provide practical insights for management personnel or any employee (e.g., managers, supervisors, and safety officers) who may want to enhance workers’ safety behaviour. Several practical interventions can be conducted to enhance remote oil and gas workers’ safety behaviour and promote an effective balance in their mental health and level of fatigue. Additionally, the findings could suggest strategies or guidelines promoting safety behaviour by focusing on psychological factors, especially those related to occupational stress, mental health and fatigue.

Occupational stressors (i.e., responsibilities towards family and living environment) have a direct negative influence on safety participation behaviour. However, there are several ways to cope with the factors that may lead to stress in life or work. According to a study on Indian workers, institutionalising spirituality in the workplace should be promoted [101]. This requires higher management to develop a holistic and comprehensive spiritual climate through practices in the organisation’s overall vision, mission, and policies. Although the study sample included managers and supervisors [101] (dissimilar to this study’s sample of workers), it is worth noting that, in the present study, the majority of the sampled population (76.2%) were Indian. Spirituality is defined as an unlimited series of personal drives, behaviours, values, experiences and attitudes that aim to encourage existential understanding, meaning and purpose [102].

Further, a leading study in stressful workplaces identified three primary strategies for reducing stress: (1) managers should attempt to remove obstacles to assist their employees, (2) workers should improve their transaction with the environment and (3) stressful relationships between individuals should be identified to assist in reducing their tension [103].

The results demonstrate that the living environment stressor could lead to poor mental health (i.e., higher levels of anxiety and depression) and higher levels of fatigue (i.e., both physical and mental), which in turn may decrease workers’ safety participation. Providing free wi-fi in the workers’ camp could be an option to encourage communication, which is the key to reduce workers’ isolation and improve their mental health. Another recommendation is that companies organise recreational facilities to be available for workers in the camp so that workers can be involved in social and sporting activities on their days off. In general, the company could offer consultations or workshops to assist the workers in coping with stress and alleviate its related anxiety and depression.

Moreover, it is suggested that boosting workers’ resilience could buffer the effects of stressors and adversity when a stressful life event occurs [104]. Resilience is considered a result of the adaptive response to a stressor, which enables individuals to cope with stressful conditions [105].

## 5. Conclusions

The study results have demonstrated that stressors affect workers’ safety participation negatively, while mental health and fatigue can act and operate as risk factors. The results show that both mental health factors (anxiety and depression) and fatigue factors (physical fatigue and mental fatigue) fully mediate the relationship between occupational stressors (responsibilities towards family and the living environment) and safety participation. However, no mediation factors significantly mediated the relationship between the occupational stressors and safety compliance. This study provides the necessary empirical evidence, which is useful for this sector or any related sector, to focus on steps to alleviate workers’ fatigue level and mental illness, as well as their stress at the workplace. The implications of these results for occupational stressors, mental health, fatigue issues and/or safety participation interventions in the industry have been discussed.

### Limitations

There are a number of limitations within the current study. Although consistent with many findings from other studies, this study focused on a sample of foreign employees working at remote oil and gas field sites in Kuwait. Therefore, any generalisations must be approached with caution. It is recommended that future research study be undertaken into other industries, contexts, and populations to compare and expand the knowledge of the effects of stressors on safety participation among remote oil and gas workers. In addition, the study’s cross-sectional data cannot adequately be used to describe and assess the causal relations between the study variables. However, the study hypotheses were formed based on reliable theoretical and literature reviews. The primary reason for adopting a cross-sectional design was the time constraints of this study. Thus, a longitudinal design to highly reflect the causal processes among the study variables is recommended.

The results from Harman’s single factor test demonstrated that CMV was not a significant concern in this study. However, the use of self-reported measures, in which responses could be inflated because the respondents tended to answer consistently, indicates that the responses have an element of subjectivity. For example, when safety behaviour is assessed by self-report, the behaviour is not tested objectively, but instead, subjectively (perceived safety behaviours), and thus, the actual safety behaviours are not observed. It is worth mentioning that the research time available was also a restriction in adding other variables that could be examined to determine their effect on general safety behaviour, such as cultural differences and culture shock (remoteness variables) as well as other occupational stressors that can cause workplace stress. For instance, the analysis of mental health was limited to anxiety and depression; other effects such as personality disorders or substance abuse were not considered. The authors selected the variables in this study to facilitate a discussion suitable for the research time available.

## Figures and Tables

**Table 1:** Scale references.

Construct	Variable	Scale	Supporting Literature
Occupational Stressors	Responsibilities towards family	-	[11,12]
	Living environment	-	
Mental Health	Anxiety	DASS21	[52]
		HADS	[53,54]
		HSCL-25	[55,56]
	Depression	DASS21	[52]
		HSCL-25	[55,56]
		CES-D	[57]
Fatigue	Physical fatigue	FAS	[58,59]
		FQ	[60,61,62]
	Mental fatigue	FAS	[58,59]
		FQ	[60,61,62]
		MFI	[63]
Safety Behaviour	Safety compliance	-	[64]
		-	[65]
		-	[66]
	Safety participation	-	[67]
		-	[65]
		-	[66]

**Table 2:** Demographic characteristics of the participants (*N* = 387).

Demographic Characteristics	Frequency	Percent%
Nationality		
Indian	295	76.2
Egyptian	35	9.0
Filipino	20	5.2
Thai	15	3.9
Others	22	5.7
Age (years)		
≤20	1	0.3
20–29	75	19.4
30–39	164	42.4
40–49	106	27.4
50–59	36	9.3
≥60	5	1.3
Marital Status		
Single	76	19.6
Married/Living with a partner	311	80.4
Last Trip Back Home		
1 month ago	152	39.0
3 months ago	57	14.7
6 months ago	68	17.6
1 year ago	83	21.4
2 years ago	24	6.2
Others	4	1.0
Days off/Weekends		
At Camp	301	77.8
Off Camp	86	22.2

**Table 3:** Anxiety mediates the relationship between responsibilities towards family and safety participation.

Regression Path	B	P	LLCI	ULCI
Path A (RTF to ANX)	0.211	<0.001	0.110	0.311
Path B (ANX to SFP)	−0.204	<0.001	−0.279	−0.129
Path C (total effect of RTF on SFP)	−0.105	<0.01	−0.183	−0.027
Path C’ (direct effect of RTF on SFP)	−0.062	=0.113	−0.139	0.015
Path A ∗ B (indirect effect of the RTF on the SFP through the ANX)	−0.043		−0.070	−0.019
Total effects R^2^ = 1.2%, F(2, 384) = 17.98, *p* < 0.001				

**Table 4:** Anxiety mediates the relationship between living environment and safety behaviour.

Regression Path (LE, ANX, SFC)	B	P	LLCI	ULCI
Path A (LE to ANX)	0.416	<0.001	0.316	0.515
Path B (ANX to SFC)	−0.210	<0.001	−0.293	−0.128
Path C (total effect of LE on SFC)	−0.044	=0.302	−0.129	0.040
Path C’ (direct effect of LE on SFC)	0.043	=0.344	−0.046	0.132
Path A ∗ B (indirect effect of the LE on the SFC through the ANX)	−0.087		−0.133	−0.051
Total effects R^2^ = 0.1%, F(2, 384) = 13.09, *p* < 0.001				
Regression Path (LE, ANX, SFP)	B	P	LLCI	ULCI
Path A (LE to ANX)	0.416	<0.001	0.316	0.515
Path B (ANX to SFP)	−0.211	<0.001	−0.291	−0.131
Path C (total effect of LE on SFP)	−0.104	<0.05	−0.186	−0.021
Path C’ (direct effect of LE on SFP)	−0.016	=0.713	−0.102	0.070
Path A ∗ B (indirect effect of the LE on the SFP through the ANX)	−0.088		−0.131	−0.052
Total effects R^2^ = 1.5%, F(2, 384) = 16.69, *p* < 0.001				

**Table 5:** Depression mediates the relationship between occupational stressors and safety behaviour.

Regression Path (RTF, DPR, SFC)	B	P	LLCI	ULCI
Path A (RTF to DPR)	0.260	<0.001	0.170	0.350
Path B (DPR to SFC)	−0.158	=0.001	−0.289	−0.134
Path C (total effect of RTF on SFC)	0.039	=0.345	−0.042	0.119
Path C’ (direct effect of RTF on SFC)	0.080	=0.059	−0.003	0.162
Path A ∗ B (indirect effect of the RTF on the SFC through the DPR)	−0.0410		−0.072	−0.018
Total effects R^2^ = −0.6%, F(2, 384) = 6.62, *p* < 0.001				
Regression Path (RTF, DPR, SFP)	B	P	LLCI	ULCI
Path A (RTF to DPR)	0.260	<0.001	0.170	0.350
Path B (DPR to SFP)	−0.096	<0.05	−0.182	−0.009
Path C (total effect of RTF on SFP)	−0.105	<0.01	−0.182	−0.027
Path C’ (direct effect of RTF on SFP)	−0.080	=0.051	−0.161	0.001
Path A* B (indirect effect of the RTF on the SFP through the DPR)	−0.025		−0.483	−0.004
Total effects R^2^ = 0.8%, F(2, 384) = 5.95, *p* < 0.01				

**Table 6:** Depression mediates the relationship between living environment and safety behaviour.

Regression Path (LE, DPR, SFC)	B	P	LLCI	ULCI
Path A (LE to DPR)	0.348	<0.001	0.255	0.440
Path B (DPR to SFC)	−0.135	<0.001	−0.226	−0.044
Path C (total effect of LE on SFC)	−0.044	=0.302	−0.129	0.040
Path C’ (direct effect of LE on SFC)	0.002	=0.958	−0.087	0.092
Path A ∗ B (indirect effect of the LE on the SFC through the DPR)	−0.047		−0.081	−0.019
Total effects R^2^ = 0.3%, F(2, 384) = 4.78, *p* < 0.01				
Regression Path (LE, DPR, SFP)	B	P	LLCI	ULCI
Path A (LE to DPR)	0.384	<0.001	0.255	0.440
Path B (DPR to SFP)	−0.094	<0.05	−0.183	−0.005
Path C (total effect of LE on SFP)	−0.104	<0.05	−0.186	−0.021
Path C’ (direct effect of LE on SFP)	−0.071	=0.111	−0.159	0.017
Path A ∗ B (indirect effect of the LE on the SFP through the DPR)	−0.033		−0.062	−0.005
Total effects R^2^ = 0.9%, F(2, 384) = 5.26, *p* < 0.01				

**Table 7:** Physical fatigue mediates the relationship between responsibilities towards family and safety behaviour.

Regression Path (RTF, PFT, SFC)	B	P	LLCI	ULCI
Path A (RTF to PFT)	0.304	<0.001	0.201	0.407
Path B (PFT to SFC)	−0.144	<0.001	−0.221	−0.067
Path C (total effect of RTF on SFC)	0.039	=0.345	−0.042	0.119
Path C’ (direct effect of RTF on SFC)	0.082	=0.05	0.000	0.165
Path A ∗ B (indirect effect of the RTF on the SFC through the PFT)	−0.044		−0.077	−0.018
Total effects R^2^ = −0.7%, F(2, 384) = 7.17, *p* = 0.001				
Regression Path (RTF, PFT, SFP)	B	P	LLCI	ULCI
Path A (RTF to PFT)	0.304	<0.001	0.201	0.407
Path B (PFT to SFP)	−0.111	<0.01	−0.186	−0.035
Path C (total effect of RTF on SFP)	−0.105	<0.01	−0.183	−0.027
Path C’ (direct effect of RTF on SFP)	−0.072	=0.082	−0.152	0.009
Path A ∗ B (indirect effect of the RTF on the SFP through the PFT)	−0.043		−	−0.019
Total effects R^2^ = 1.0%, F(2, 384) = 7.76, *p* < 0.001				

**Table 8:** Physical fatigue mediates the relationship between living environment and safety behaviour.

Regression Path (LE, PFT, SFC)	B	P	LLCI	ULCI
Path A (LE to PFT)	0.421	<0.001	0.307	0.517
Path B (PFT to SFC)	−0.124	<0.01	−0.204	−0.044
Path C (total effect of LE on SFC)	−0.044	=0.302	−0.129	0.040
Path C’ (direct effect of LE on SFC)	0.007	=0.884	−0.083	0.097
Path A ∗ B (indirect effect of the LE on the SFC through the PFT)	−0.087		−0.133	−0.051
Total effects R^2^ = 0.3%, F(2, 384) = 5.20, *p* < 0.01				
Regression Path (LE, PFT, SFP)	B	P	LLCI	ULCI
Path A (LE to PFT)	0.412	<0.001	0.317	0.517
Path B (PFT to SFP)	−0.111	<0.001	−0.189	−0.033
Path C (total effect of LE on SFP)	−0.104	<0.05	−0.186	−0.021
Path C’ (direct effect of LE on SFP)	−0.058	=0.194	−0.146	0.030
Path A ∗ B (indirect effect of the LE on the SFP through the PFT)	−0.046		−0.079	−0.015
Total effects R^2^ = 1.1%, F(2, 384) = 7.07, *p* = 0.001				

**Table 9:** Mental fatigue mediates the relationship between responsibilities towards family and safety behaviour.

Regression Path (RTF, MFT, SFC)	B	P	LLCI	ULCI
Path A (RTF to MFT)	0.309	<0.001	0.201	0.407
Path B (MFT to SFC)	−0.239	<0.001	−0.321	−0.158
Path C (total effect of RTF on SFC)	0.039	=0.345	−0.042	0.119
Path C’ (direct effect of RTF on SFC)	0.113	<0.01	0.031	0.194
Path A ∗ B (indirect effect of the RTF on the SFC through the MFT)	−0.074		−0.112	−0.044
Total effects R^2^ = −1.5%, F(2, 384) = 17.15, *p* < 0.001				
Regression Path (RTF, MFT, SFP)	B	P	LLCI	ULCI
Path A (RTF to MFT)	0.309	<0.001	0.204	0.404
Path B (MFT to SFP)	−0.211	<0.01	−0.291	−0.131
Path C (total effect of RTF on SFP)	−0.105	<0.01	−0.183	−0.027
Path C’ (direct effect of RTF on SFP)	−0.040	=0.322	−0.120	0.039
Path A ∗ B (indirect effect of the RTF on the SFP through the MFT)	−0.065		−0.097	−0.039
Total effects R^2^ = 1.6%, F(2, 384) = 13.31, *p* < 0.001				

**Table 10:** Mental fatigue mediates the relationship between living environment and safety behaviour.

Regression Path (LE, MFT, SFC)	B	P	LLCI	ULCI
Path A (RTF to ANX)	0.211	<0.001	0.110	0.311
Path B (ANX to SFC)	−0.212	<0.001	−0.289	−0.134
Path C (total effect of RTF on SFC)	0.039	=0.345	−0.042	0.119
Path C’ (direct effect of RTF on SFC)	0.083	<0.05	0.004	0.163
Path A ∗ B (indirect effect of the RTF on the SFC through the ANX)	−0.0446		−0.077	−0.020
Total effects R^2^ = −0.8%, F(2, 384) = 14.88, *p* < 0.001				
Regression Path (LE, MFT, SFP)	B	P	LLCI	ULCI
Path A (LE to MFT)	0.461	<0.001	0.366	0.555
Path B (MFT to SFP)	−0.223	<0.001	−0.308	−0.139
Path C (total effect of LE on SFP)	−0.104	<0.05	−0.186	−0.021
Path C’ (direct effect of LE on SFP)	−0.001	=0.984	−0.090	0.088
Path A ∗ B (indirect effect of the LE on the SFP through the MFT)	−0.103		−0.143	−0.066
Total effects R^2^ = 1.6%, F(2, 384) = 16.78, *p* < 0.001				

## Data Availability

The data that support the findings of this study are available on request from the corresponding author, [A.S.A.]. The data are not publicly available due to [restrictions e.g. their containing information that could compromise the privacy of research participants].
